# Dynamic Thermography-Based Early Breast Cancer Detection Using Multivariate Time Series

**DOI:** 10.3390/s25247649

**Published:** 2025-12-17

**Authors:** María-Angélica Espejel-Rivera, Carina Toxqui-Quitl, Alfonso Padilla-Vivanco, Raúl Castro-Ortega

**Affiliations:** Computer Vision Laboratory, Universidad Politécnica de Tulancingo, Hidalgo 43629, Mexico; carina.toxqui@upt.edu.mx (C.T.-Q.); alfonso.padilla@upt.edu.mx (A.P.-V.); raul.castro@upt.edu.mx (R.C.-O.)

**Keywords:** heat source parameters, time series classification, infrared imaging, dynamic thermography, breast cancer, D-I-R model, medical image classification

## Abstract

A computational approach for early breast cancer detection using Dynamic Infrared Thermography (DIT) was developed. Thermograms are represented by multivariate time series extracted from thermal hotspots in the breast, capturing five features: maximum and mean temperature, spatial heterogeneity, heat flux, and tumor depth, over 20 thermograms. Features are estimated based on the inverse solution of the Pennes bio-heat equation. Classification is performed using a Time Series Forest (TSF) and a Long Short-Term Memory (LSTM) network. The TSF achieved an accuracy of 86%, while the LSTM reached 94% accuracy. These results indicate that dynamic thermal responses under cold-stress conditions reflect tumor angiogenesis and metabolic activity, demonstrating the potential of combining multivariate thermographic sequences, biophysical modeling, and machine learning for non-invasive breast cancer screening.

## 1. Introduction

Breast cancer remains one of the most prevalent and impactful cancers worldwide. According to the Global Cancer Observatory (GCO) [1] and related cancer research organizations, the incidence of breast cancer has been increasing globally, including in various countries such as Mexico. In 2022, it was estimated that there were approximately 2.3 million new cases of breast cancer and around 685,000 deaths worldwide. Reducing breast cancer involves a multifaceted approach that includes prevention, early detection, lifestyle modifications, and access to treatment. Several methods are used to detect cancer [2]. Xiao J. et al. developed an innovative pipeline that used targeted proteomics and metabolomics to identify potential biomarkers for early detection of Hepatocellular carcinoma [3]. Grosmanova E. et al. focused on precise visualization of tumor boundaries using HPMA copolymers [4]. Lawson and Chughtai [5] reported using surface temperature measurements as a viable tool for breast cancer detection. As stated above, diseases alter the thermal gradient in this area and subtle thermal abnormalities can be associated with specific disorders. Therefore, research on medical applications of infrared technology has been published and different databases have been created, such as the public DMR-IR [6,7]. Breast cancer detection using thermography has evolved from static imaging to dynamic and computationally enhanced approaches that integrate experimental measurements, advanced modeling, and machine learning. Research in this domain can be categorized into five complementary methodological groups: (i) foundational thermography: passive and active approaches, (ii) dynamic infrared thermography and temporal analysis, (iii) machine learning for thermal image interpretation, (iv) vascular and perfusion assessment via thermography, (v) computational modeling and patient-specific simulations.

### 1.1. Foundational Thermography: Passive and Active Approaches

Passive and active thermography represent foundational techniques for breast cancer detection. Passive thermography captures natural thermal emissions from the breast surface, providing insight into superficial metabolic and vascular activity. Active thermography applies controlled thermal stimuli, such as localized cooling or heating, to enhance thermal contrast and reveal deeper perfusion patterns. Jacob et al. [8] demonstrated that active thermography improves tumor detectability, increasing the effective depth from approximately 3 mm (passive) to 9 mm (active). They also emphasized that device resolution, imaging protocols, and image-processing methods strongly influence diagnostic performance, and that thermography remains a complementary tool alongside mammography and ultrasound.

### 1.2. Dynamic Infrared Thermography and Temporal Analysis

DIT extends conventional thermography by analyzing temporal changes in skin temperature following external perturbations. Gershenson et al. [9,10] applied Principal Component Analysis (PCA) and Independent Component Analysis (ICA) to sequences of thermal images, successfully separating signals associated with cancer-related heat, vasomodulation, and superficial perfusion. Virtual wave transformations further enabled detailed analysis of vascular responses, suggesting that vasoconstriction patterns may correlate with malignancy. Gonzalez et al. [11] highlighted the integration of numerical simulations, automatic feature extraction, and artificial intelligence to improve sensitivity and specificity. Salhab et al. [12] demonstrated that DIT enables real-time monitoring of metabolic activity and angiogenesis, providing early detection biomarkers that often precede structural changes detectable by mammography. Bandyopadhyay et al. [13] introduced rotational infrared imaging, combined with machine learning, to capture high-quality images from multiple perspectives, thereby enhancing screening coverage.

### 1.3. Machine Learning for Thermal Image Interpretation

Machine learning techniques have become essential for automated interpretation of thermal images. Gayoumi et al. [14] combined dynamic thermography with deep auto-encoder neural networks, extracting statistical features from sequential images and achieving 94.87% accuracy and 96.77% specificity on a 196-subject dataset. Ekici et al. [15] integrated infrared imaging with convolutional neural networks, reaching nearly 99% accuracy. Silva et al. [16] developed a framework extracting texture descriptors—Local Binary Patterns (LBP) and Haralick features—from thermal recovery sequences to quantify tissue heterogeneity, which were then classified using a k-Nearest Neighbors (k-NN) algorithm. Alzahrani et al. [17] enhanced CNN-based classification with particle swarm optimization, enabling accurate discrimination between malignant and benign thermographic images. These studies demonstrate that machine learning can effectively leverage dynamic thermal and textural data for reliable lesion characterization.

Although most recent deep learning studies apply CNNs or transformer architectures directly to thermographic images, these image-based approaches differ fundamentally from the methodology adopted in the present work. In contrast to pixel-level classification, our framework extracts physiologically meaningful descriptors from a Region of Interest (RoI) and analyzes their temporal evolution over a 20-frame dynamic sequence. This representation transforms breast thermography into a multivariate time series classification problem, enabling models such as LSTM and TSF to capture rewarming dynamics, metabolic heat generation, and spatial heterogeneity rather than static textural patterns. As discussed later in the manuscript, this physiologically grounded approach achieves performance comparable to state-of-the-art image-based deep learning models while offering enhanced interpretability and suitability for small-sample dynamic datasets.

### 1.4. Vascular and Perfusion Assessment via Thermography

Infrared thermography has also been applied to assess vascular function and skin perfusion. Lozano et al. [18] developed a patient-specific thermal model combining infrared (IR) imaging, 3D breast scans, and magnetic resonance imaging (MRI) to estimate perfusion and metabolic heat, finding markedly higher heat generation in tumors. Du et al. [19] studied transient skin temperature responses to step changes in environmental conditions, revealing correlations between heat loss and thermal sensation, which informs the interpretation of dynamic thermal signals. These studies highlight the potential of thermography to quantify vascular responses and provide physiologically relevant information.

### 1.5. Computational Modeling and Patient-Specific Simulations

Computational modeling provides a framework for understanding the physiological mechanisms underlying thermographic observations. Chanmugan et al. [20] developed a 3D finite element model of the breast to investigate the impact of tumor size, depth, metabolic heat, and perfusion on surface temperature. Nowakowski et al. [21] combined infrared thermography with thermal modeling to reconstruct internal tissue structures, capturing dynamic temperature changes synchronized with external stimulation. Pérez Raya [22] proposed a patient-specific thermal modeling approach using physics-based artificial intelligence to analyze breast thermal fields without requiring complex simulation software. Collectively, these model-based approaches enable non-invasive and quantitative estimation of physiological parameters related to breast heat transfer and perfusion, thereby providing a foundation for integrating thermography with dynamic analysis and machine learning techniques.

**Figure 1:**
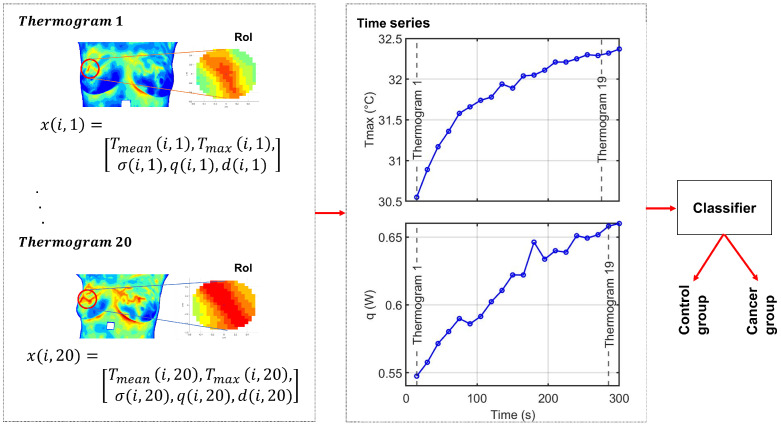
Overall experimental framework of the proposed method. Each thermogram t∈{1,…,M} represents a time point in the dynamic sequence. For subject *i*, the feature vector at time *t* is $x_{i,t}={\left[T_{\max}\left(i,t\right),T_{\mathrm{mean}}\left(i,t\right),\sigma \left(i,t\right),q\left(i,t\right),d\left(i,t\right)\right]}^{⊤}\in R^{V}$. The full sequence of *M* thermograms forms the multivariate time series $X_{i}\in R^{M\times V}$, from which five univariate time series, one per feature, are derived for classification. The color scale represents temperature variations, with red indicating higher temperatures and blue indicating lower temperatures.

Despite these advances, challenges remain regarding data quality, interpretability, and scalability, especially in biomedical contexts [23]. In this work, we propose a complementary dynamic-thermography framework that models breast thermal behaviour through physiologically grounded temporal descriptors extracted from a RoI. Building upon these multivariate time series representations, we evaluate two machine learning classifiers, Time Series Forest (TSF) and Long Short-Term Memory (LSTM) networks, to assess their ability to discriminate between cancer and control cases using dynamic thermographic information. An overview of the methodological pipeline is provided in Figure 1. This approach aims to enhance diagnostic performance while improving the interpretability of machine learning predictions in breast thermography. The manuscript is organized as follows. Section 2 describes the methodological framework, including the estimation of physiological parameters from thermographic data, the construction of multivariate time series representations, and the TSF and LSTM classification models. Section 3 presents the experimental setup and results, including the description of the DMR-IR database, the quantitative analysis of thermal recovery under cold stress, and the classification experiments performed on multivariate time series derived from 50 dynamic breast thermogram sequences. Finally, Section 4 discusses the main findings, clinical implications, and methodological contributions of this work, and provides concluding remarks.

## 2. Methods

### 2.1. Heat Source Model: A Mathematical Review

The thermal transfer/heat conduction equation of a specimen is given as [24],

(1) $$\rho C_{p}\frac{\partial T}{\partial t}=k\frac{\partial^{2}T}{\partial t^{2}}+q$$

where T=T(x,y,z) is a temperature field, *k* is the thermal conductivity constant from the material (W/m/C), ρ is the density ($Kg/m^{3}$), $C_{p}$ is specific heat (J/KgC), and q(x,y,z,t) is the internal heat generation function per unit volume. A transient heat conduction equation that accounts for the metabolic heat generated within the tissue and the heat transfer between the tissue and the blood is the Pennes bio-heat equation and it is given as [24],

(2) $$\rho_{t}c_{t}\frac{\partial T_{t}}{\partial t}=▽\left(k_{t}\cdot ▽T_{t}\right)+w_{b}\rho_{b}c_{b}\left(T_{a}-T_{t}\right)+q_{m}.$$

where $w_{b}$ represents the flow rate of blood, and *b*, and *a* in Equation (2), the additive term stands for blood and arteries, respectively. The terms $w_{b},\rho_{b}c_{b}\left(T_{a}-T\right)+q_{m}$ are merged to be the internal heat source. A solution of Equation (2) is given by [25,26,27],

(3) $$T=T_{e}+\frac{q}{4\pi h_{0}r^{2}}.$$

**Figure 2:**
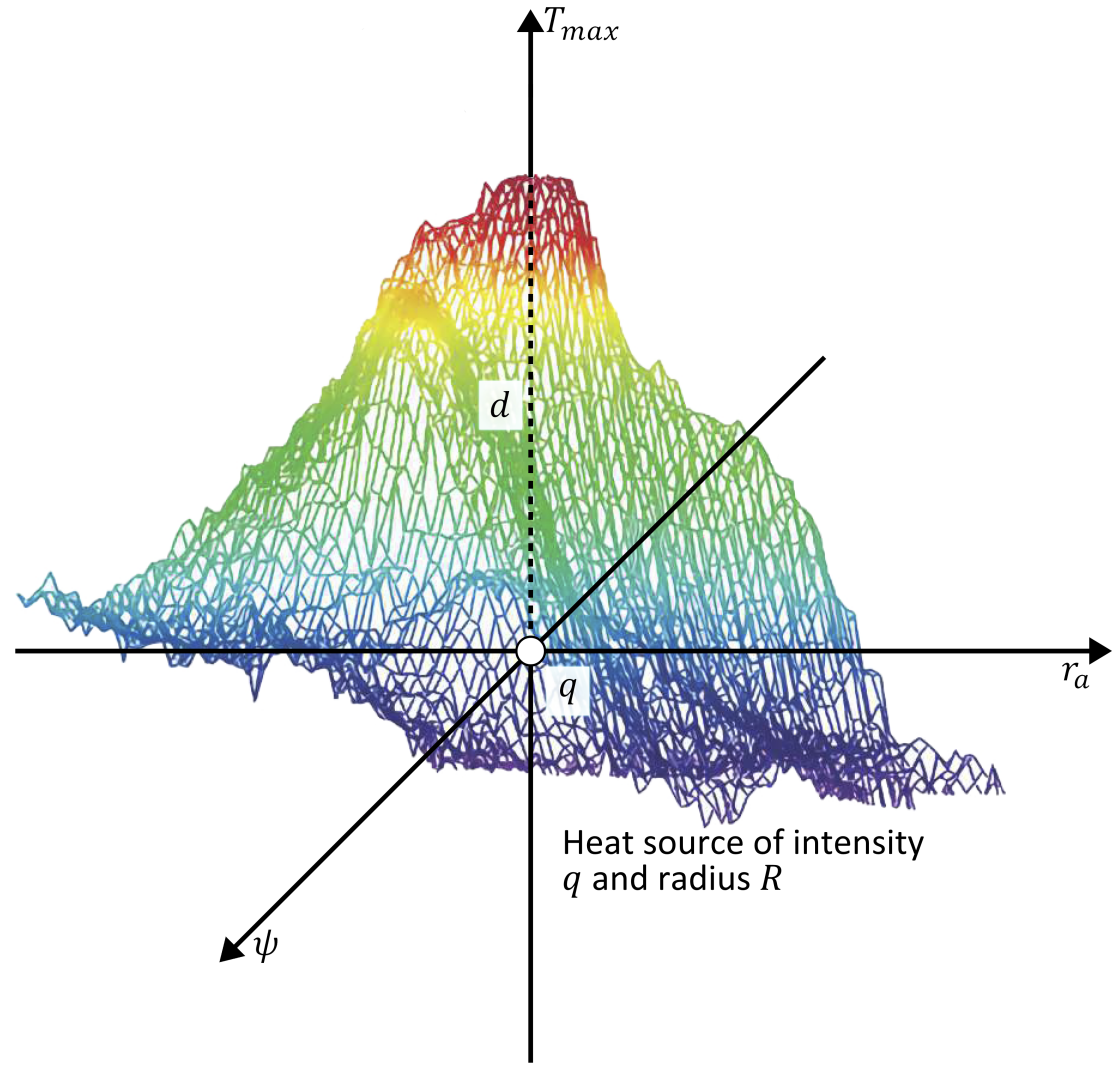
Schematic representation of the theoretical model of an internal heat source characterized by depth *d*, intensity *q*, and radius *R*. The color scale represents temperature variations, with red indicating higher temperatures and blue indicating lower temperatures.

The maximum temperature $T_{max}$ is obtained when $r_{a}=0$, which is the temperature at the center point in Figure 2. Suppose *a* is the distance from the origin to an arbitrary point on body surface, then $r^{2}=d^{2}+a^{2}$. Therefore,

(4) $$T\left(a\right)=T_{e}+\frac{q}{4\pi h_{0}\left(d^{2}+a^{2}\right)}.$$

Abnormal tissue can be modeled as a spherical heat source with intensity *q*, radius *R*, and depth *d* [25,28]. Then,

(5) $$T\left(a\right)=T_{e}+\frac{q}{4\pi h_{0}\left[{\left(d+R\right)}^{2}+a^{2}\right]},$$

where T(a) is the temperature at any arbitrary point *a* on the STD of the thermal input data. The temperature distribution T(a) is obtained from the thermal input data at each side of the maximum temperature point $T_{max}$.

#### D-I-R Model

The heat source parameters are obtained through the D-I-R model as [25],

(6) $$d\left(a\right)=\frac{a\sqrt{\left(T\left(a\right)-T_{e}\right)}}{\sqrt{T_{max}-T\left(a\right)}},$$

(7) $$q\left(a\right)=4\pi h_{0}\frac{\left(T\left(a\right)-T_{e}\right)\left(T_{max}-T_{e}\right)}{T_{max}-T\left(a\right)}a^{2},$$

and

(8) $$R=\sqrt[{3}]{\frac{q}{Q_{m}A_{t}}},$$

for

$Q_{m}=418.6$ *W*/*m*^3^, $h_{0}=8.77$ *W*/$m^{2}$·°C, and volume of cell is *A_t_* = 1 μm [25]. To quantify the effect of each physiological parameter, the D-I-R model is simulated.

In each time frame *t*, the radial temperature profile T(a) is extracted from the hottest pixel outwards, and the parameters q(t) and d(t) are calculated using a fixed radial distance a=0.0168 m (approximately 1.7 cm), following the original validation reported in [29]. The classification step makes use of the physiological pattern vectors $x_{i,t}=\left\{T_{\max}\left(i,t\right),T_{\mathrm{mean}}\left(i,t\right),\sigma \left(i,t\right),q\left(i,t\right),d\left(i\right)\right\}$.

### 2.2. Mathematical Modeling of Thermal Recovery

The thermal recovery process in biological tissues during dynamic thermography can be described by a modified Newton’s Law of Cooling [30], which accounts for the physiological response of tissue to thermal stress. To quantitatively characterize the recovery kinetics, the temperature evolution of the tissue was modeled using the exponential function proposed by [31]:

(9) $$T\left(t\right)=T_{\infty }-\left(T_{\infty }-T_{o}\right)\cdot e^{-t/\tau }$$

where:

- T(t): tissue surface temperature at time *t* after cooling, (°C);
- $T_{o}$: initial temperature immediately after cold stress, (°C);
- $T_{\infty }$: asymptotic temperature as time →∞, (°>C);
- τ: thermal recovery time constant (s).

This model allows for the extraction of the thermal recovery time constant τ, which serves as a quantitative descriptor of tissue response and can be subsequently used for comparison between different tissue types.

### 2.3. Time Series Classification

Time series classification (TSC) is a machine learning task where a model is trained on labeled time series and subsequently used to predict the class of unseen sequences [32,33]. The temporal ordering of values is a key characteristic in this problem. Classification approaches can be broadly categorized into four main types: feature-based, distance-based, interval-based, and deep learning [33,34,35,36].

Feature-based methods extract descriptors, such as statistical moments, extrema, or Fourier and Wavelet coefficients, which are then provided to conventional classifiers. Distance-based methods rely on similarity metrics, most notably dynamic time warping (DTW), and classify using algorithms such as *k*-nearest neighbors (KNN) or support vector machines (SVM). Interval-based methods divide a series into random intervals, compute features such as mean, standard deviation, and slope, and then train classifiers on the resulting feature vectors [37]. Deep learning models, including Convolutional Neural Networks (CNN), LSTM networks, and Generative Adversarial Networks (GAN), can automatically learn complex representations from raw data [38,39,40]. LSTMs, in particular, have shown superior performance in biomedical applications, achieving state-of-the-art results across extensive benchmark collections [40,41,42]. In this work, we focus on the TSF classifier and LSTM-based neural networks.

### 2.4. Multivariate Time Series

Each person in our study is represented by a multivariate time series extracted from a RoI within the dynamic thermogram sequence. The RoI corresponds to thermal hotspots located in breast regions with higher cancer prevalence.

**Figure 3:**
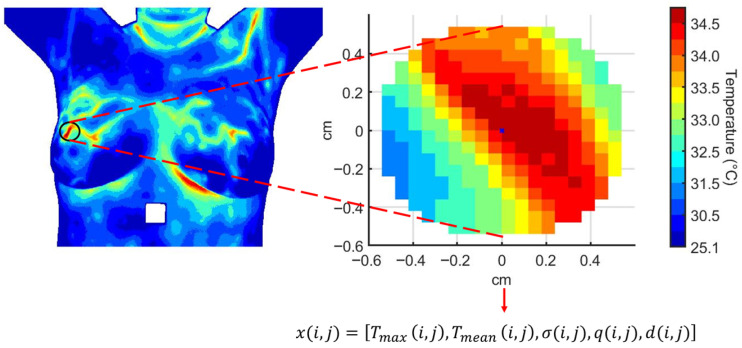
Multivariate time series extracted from a region of interest over the thermographic sequence. Each feature evolves along the temporal sequence of thermograms as $\left\{T_{\max}^{\left(i\right)}\left(t\right)\right\}$, $\left\{T_{\mathrm{mean}}^{\left(i\right)}\left(t\right)\right\}$, $\left\{\sigma^{\left(i\right)}\left(t\right)\right\}$, $\left\{q^{\left(i\right)}\left(t\right)\right\}$, and $\left\{d^{\left(i\right)}\left(t\right)\right\}$ for person *i*.

At each thermogram *t* in the dynamic sequence, the feature vector for person *i* is defined as

(10) $$x_{i,t}=\left[\begin{array}{c} T_{\max}\left(i,t\right)\\ T_{\mathrm{mean}}\left(i,t\right)\\ \sigma (i,t)\\ q(i,t)\\ d(i,t) \end{array}\right]\in R^{V},$$

where i∈{1,…,N} indexes the person, t∈{1,…,M} indexes the thermograms (M=20), and V=5 is the number of extracted thermal features: $T_{\max},\;T_{\mathrm{mean}},\;\sigma ,\;q,\;d$ (Figure 3).

The complete dataset is represented as a three-dimensional tensor:

(11) $$X\in R^{N\times M\times V},$$

where N=50 patients, M=20 thermograms per person forming the temporal sequence, and V=5 extracted features. For a single person *i*, the sequence of thermograms is organized as

$$X_{i}=[\begin{array}{cccc} x_{i,1} & x_{i,2} & \ldots & x_{i,M} \end{array}]\in R^{M\times V}.$$

From the multivariate sequence $X_{i}$, we can extract five univariate time series, one for each feature, representing their evolution across the temporal sequence:

$$\begin{array}{cc} T_{\max}^{\left(i\right)} & ={\left[T_{\max}\left(i,1\right),T_{\max}\left(i,2\right),\ldots ,T_{\max}\left(i,M\right)\right]}^{⊤},\\ T_{\mathrm{mean}}^{\left(i\right)} & ={\left[T_{\mathrm{mean}}\left(i,1\right),T_{\mathrm{mean}}\left(i,2\right),\ldots ,T_{\mathrm{mean}}\left(i,M\right)\right]}^{⊤},\\ \sigma^{\left(i\right)} & ={\left[\sigma (i,1),\sigma (i,2),\ldots ,\sigma (i,M)\right]}^{⊤},\\ q^{\left(i\right)} & ={\left[q(i,1),q(i,2),\ldots ,q(i,M)\right]}^{⊤},\\ d^{\left(i\right)} & ={\left[d(i,1),d(i,2),\ldots ,d(i,M)\right]}^{⊤}. \end{array}$$

Each series captures the temporal evolution of a single thermal feature for patient *i* across the dynamic sequence. Together, these five univariate series constitute the multivariate time series $X_{i}$, which serves as input for time series classification models.

### 2.5. Time Series Forest Classifier

TSF is an ensemble-based classifier that extends the Random Forest paradigm to time series data [37]. It transforms each univariate or multivariate time series into interval-based summary features, which are then used to train decision trees. This approach efficiently captures local temporal patterns while remaining computationally tractable and interpretable.

#### 2.5.1. Feature Extraction

For a univariate time series $x_{i,v}={\left[x_{i,v}\left(1\right),\ldots ,x_{i,v}\left(M\right)\right]}^{⊤}$, TSF samples multiple intervals [s,e] along the temporal axis, with 1≤s<e≤M and $L=e-s+1\geq \ell_{\min}$, where $\ell_{\min}$ is the minimum interval length. For each interval, three summary features are computed:

- Mean: $\mu_{s:e}^{\left(i,v\right)}=\frac{1}{L}\sum_{j=s}^{e}x_{i,v}\left(j\right)$;
- Standard deviation: $\sigma_{s:e}^{\left(i,v\right)}=\sqrt{\frac{1}{L-1}\sum_{j=s}^{e}{\left(x_{i,v}\left(j\right)-\mu_{s:e}^{\left(i,v\right)}\right)}^{2}}$;
- Slope: regression coefficient ${\hat{\beta }}_{s:e}^{\left(i,v\right)}$ obtained from $x_{i,v}\left(j\right)\approx \alpha +\beta j,\;j\in \left[s,e\right]$.

For a multivariate sequence $X_{i}\in R^{M\times V}$, these interval features are computed independently for each variable v∈{1,…,V}. Once all features are computed, they are concatenated into a single, fixed-length feature vector. Let *k* denote the number of sampled intervals per variable; the resulting vector has dimension 3 kV:

(12) $$f_{i}={\left[\mu_{1}^{\left(1\right)},\sigma_{1}^{\left(1\right)},\beta_{1}^{\left(1\right)},\ldots ,\mu_{k}^{\left(V\right)},\sigma_{k}^{\left(V\right)},\beta_{k}^{\left(V\right)}\right]}^{⊤}\in R^{3kV}.$$

#### 2.5.2. Ensemble Construction

**Figure 4:**
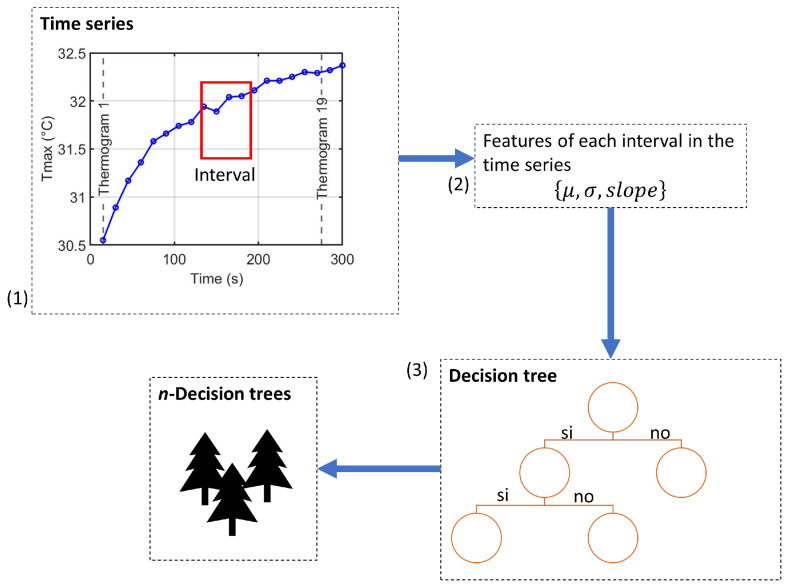
Ensemble construction process of the Time Series Forest classifier: (1) random interval selection, (2) extraction of summary features for each variable, and (3) training of decision trees using the interval-based features.

TSF builds an ensemble following the Random Forest paradigm [43], as illustrated in Figure 4.

1. For each tree, randomly select *k* intervals along the time axis of each variable.
2. Compute summary statistics {μ,σ,slope} for each interval and concatenate them into a feature vector $f_{i}^{\mathrm{tree}}$.
3. Train a decision tree on this interval-based feature representation.
4. Repeat for *n* trees to form the ensemble; final predictions are obtained by majority voting.

To illustrate the process of multivariate feature extraction, consider person *i* with thermographic sequence $X_{i}\in R^{M\times V}$ (M=20, V=5). Suppose k=2 intervals per variable for a given tree:

- Variable 1 ($T_{\max}$): [1,3], [6,8];
- Variable 2 ($T_{\mathrm{mean}}$): [2,5], [7,10];
- Variables 3–5 sampled similarly.

After computing {μ,σ,β} for each interval, features are concatenated as

$$f_{i}^{\mathrm{tree}}={\left[\mu_{1}^{\left(1\right)},\sigma_{1}^{\left(1\right)},\beta_{1}^{\left(1\right)},\ldots ,\mu_{2}^{\left(5\right)},\sigma_{2}^{\left(5\right)},\beta_{2}^{\left(5\right)}\right]}^{⊤}\in R^{3kV}=R^{30}.$$

Repeating this process for *n* trees with different random intervals enables the ensemble to capture diverse temporal patterns. Final predictions are obtained by majority voting across all trees.

TSF evaluates splits using the Entrance Gain criterion [37] and supports parallel tree training. The temporal importance curve provides interpretability by highlighting which intervals and variables contribute most to class discrimination [37,44]. This framework efficiently handles multivariate data, avoids explicit temporal alignment, and provides interpretable temporal features for automated classification into cancer group or control group.

#### 2.5.3. TSF Hyperparameters

Hyperparameters define model configuration settings specified before training, controlling bias–variance trade-off and computational efficiency [45]. For TSF, key hyperparameters include:

- Minimum interval length ($\ell_{\min}$): Shortest interval considered. Smaller values capture short-term fluctuations but increase overfitting risk and computation; typical $\ell_{\min}=3$ [37].
- Number of trees (*n*): Ensemble size; larger *n* reduces variance at higher computation cost, often n∈[100, 500] [37,46].
- Intervals per tree: Number of random intervals sampled per variable; heuristic: $\sqrt{m}$ where *m* is series length [37,43].
- Random seed and parallel jobs ($n_{\mathrm{jobs}}$): Ensure reproducibility and efficient multi-core training [43].

### 2.6. Long Short-Term Memory Networks for Time Series Classification

LSTM networks are a specialized type of Recurrent Neural Network (RNN) designed to overcome the vanishing gradient problem, enabling the modeling of long-range temporal dependencies [47]. This makes them particularly effective for TSC, where predictive patterns often span extended temporal horizons [40,48].

#### 2.6.1. Architecture and Gating Mechanisms

**Figure 5:**
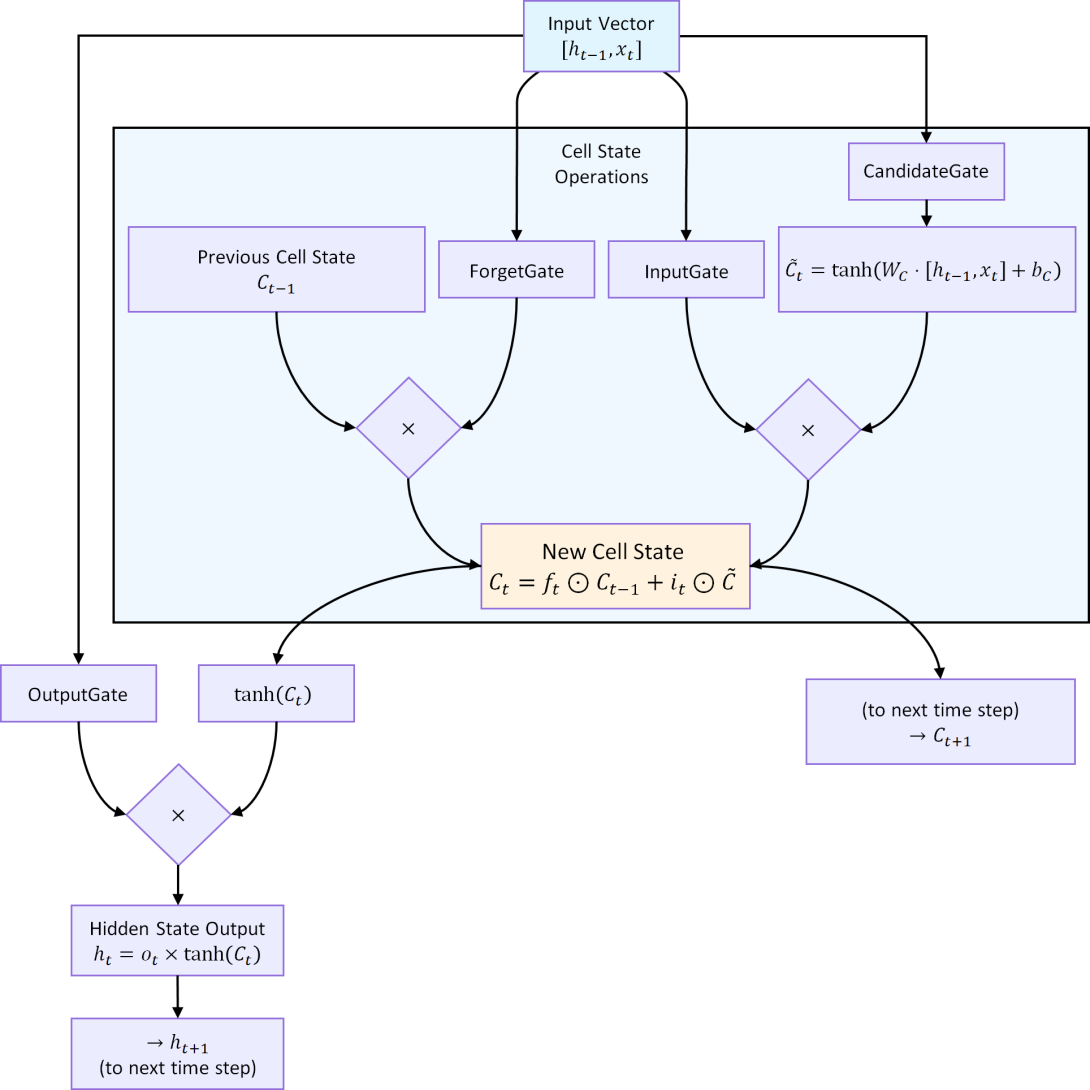
Architecture of an LSTM unit. The forget, input, and output gates regulate the flow of information, enabling selective retention and propagation of relevant temporal patterns.

An LSTM unit regulates information flow through three gates, as illustrated in Figure 5.

Forget gate:

(13) $$f_{t}=\sigma \left(W_{f}\cdot \left[h_{t-1},x_{t}\right]+b_{f}\right)$$

Input gate and candidate state:

(14) $$\begin{array}{cc} i_{t} & =\sigma (W_{i}\cdot \left[h_{t-1},x_{t}\right]+b_{i}) \end{array}$$

(15) $$\begin{array}{cc} {\tilde{C}}_{t} & =\tanh(W_{C}\cdot \left[h_{t-1},x_{t}\right]+b_{C}) \end{array}$$

Cell state update:

(16) $$C_{t}=f_{t}⊙C_{t-1}+i_{t}⊙{\tilde{C}}_{t}$$

Output gate and hidden state:

(17) $$\begin{array}{cc} o_{t} & =\sigma (W_{o}\cdot \left[h_{t-1},x_{t}\right]+b_{o}) \end{array}$$

(18) $$\begin{array}{cc} h_{t} & =o_{t}⊙\tanh\left(C_{t}\right) \end{array}$$

where ⊙ denotes element-wise multiplication, $C_{t}$ is the cell state, $h_{t}$ the hidden state, and $x_{t}$ the input at time *t*. This gating structure (Figure 5) allows selective retention, updating, and propagation of temporal information across multiple time steps.

#### 2.6.2. Application in Time Series Classification

LSTM networks are particularly effective for TSC because they preserve contextual information across extended temporal horizons. By maintaining a persistent internal state, LSTMs are able to simultaneously capture short-term fluctuations and long-term dependencies, which are often critical for distinguishing between different classes of temporal signals [41,49]. This property is especially valuable in domains such as biomedical time series, where subtle temporal variations can carry diagnostic significance.

In practical TSC tasks, LSTM variants adapt the architecture to different requirements:

- Unidirectional LSTM: Processes sequences in the forward temporal direction, making it suitable for real-time applications where only past context is available.
- Bidirectional LSTM (BiLSTM): Processes input in both forward and backward directions, thereby exploiting the full temporal context of a sequence. This typically improves classification accuracy, though at the expense of higher computational complexity [35].
- LSTM with Attention: Augments the model with a mechanism that learns adaptive weights over time steps, highlighting the most informative segments of the sequence. This not only enhances predictive performance but also improves interpretability by revealing which temporal regions drive the classification [42].

#### 2.6.3. Classification Layer and Readout Mechanisms

Once temporal features have been extracted by the LSTM layers, they must be aggregated into a final decision. The conventional approach relies on the last hidden state, which is passed to a fully connected layer with softmax activation to yield class probabilities:

(19) $$y=\mathrm{softmax}(W_{y}\cdot h_{T}+b_{y}).$$

**Figure 6:**
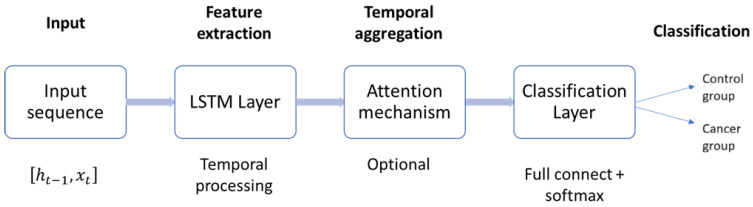
General architecture of the LSTM-based time series classifier. Input sequences are processed through stacked LSTM layers, aggregated via attention or convolutional readouts, and mapped to class probabilities by a fully connected softmax layer.

While effective, this strategy may underutilize valuable information present in earlier hidden states. To address this limitation, more advanced readout mechanisms have been developed. Attention pooling [50], for example, computes a weighted sum of hidden states, enabling the model to focus on the most discriminative time steps. Similarly, temporal convolutional readouts aggregate sequence-wide information through convolutional filters, capturing multi-scale temporal patterns before classification [40]. As illustrated in Figure 6, the general LSTM-based pipeline for time series classification consists of four stages: input processing, feature extraction via LSTM layers, temporal aggregation (with or without attention), and the final classification layer. Advanced readout mechanisms enhance the temporal aggregation stage, thereby producing more robust and well-informed classification outcomes.

#### 2.6.4. LSTM Hyperparameters

The performance of LSTM networks depends critically on their hyperparameters [45]:

- Number of layers: Deeper networks can model more complex dynamics but risk overfitting and higher computational cost. Typical values range from 1 to 3.
- Hidden units per layer: Determines the dimensionality of the hidden state $h_{t}$. Larger values increase representational power but also memory and computation; common settings range from 50 to 200 units [40].
- Sequence length (window size): Defines how many time steps are fed into the network. Longer windows capture more context but may increase noise and training complexity.
- Dropout rate: A regularization parameter that randomly deactivates units during training to prevent overfitting. Typical values are 0.2–0.5 [51].
- Learning rate: Controls step size during gradient descent optimization. Small values improve stability but slow convergence; common ranges are $10^{-4}$ to $10^{-2}$.
- Batch size and number of epochs: Batch size controls the number of sequences processed simultaneously, balancing memory usage and convergence stability. The number of epochs defines how many times the dataset is iterated during training.
- Optimizer: Adaptive algorithms such as Adam are commonly used due to their efficiency and robustness across TSC tasks [52].

### 2.7. Evaluation Metrics

To assess the performance of the proposed classifiers, three complementary metrics were employed: accuracy, F1-score, and the Area Under the ROC Curve (AUC). Together, these measures provide a comprehensive evaluation of diagnostic performance from both threshold-dependent and threshold-independent perspectives and align with widely accepted recommendations for clinical machine learning evaluation [53,54].

- Accuracy quantifies the proportion of correctly classified cases among all evaluated instances and provides an intuitive global measure of performance. Although the dataset used in this study is balanced, accuracy alone does not reveal how errors are distributed between false positives and false negatives—two misclassification types with distinct clinical implications in breast cancer screening [55].
- F1-score, defined as the harmonic mean of precision and recall, complements accuracy by capturing the trade-off between identifying malignant cases (recall or sensitivity) and avoiding unnecessary alarms (precision). This metric is particularly relevant in diagnostic applications, where sensitivity is essential to avoid missed malignancies while precision helps reduce unnecessary follow-up procedures.
- AUC provides a threshold-independent assessment of classifier separability by summarizing the receiver operating characteristic (ROC) curve, which plots sensitivity against the false-positive rate. Because AUC reflects the probability that a randomly selected cancer case will receive a higher predicted risk than a randomly selected control case, it offers a robust measure of intrinsic discriminative ability and is considered a standard performance metric in medical predictive modeling [53].

To complement these scalar metrics, we also report normalized confusion matrices for each feature set and for both classifiers (TSF and LSTM). Each matrix is computed within a 5-fold cross-validation scheme and subsequently averaged across folds. Normalization (i.e., scaling each row by the number of true samples in that class) expresses classification outcomes as rates rather than raw counts. Even in balanced datasets, this normalization is essential for clinical interpretation because it enables direct quantification of sensitivity (true positive rate) and specificity (true negative rate), two measures that directly reflect diagnostic reliability and are routinely reported in clinical model validation [54].

The normalized confusion matrices provide a threshold-dependent view of classification behavior, complementing the threshold-independent information conveyed by AUC. They allow identification of systematic tendencies—such as whether a classifier is more prone to misclassifying borderline cases as benign or malignant—that may not be apparent from aggregated metrics alone.

In summary, the combined use of accuracy, F1-score, AUC, and normalized confusion matrices yields a rigorous and clinically meaningful characterization of classifier performance. Accuracy reflects global correctness, F1-score highlights the balance between sensitivity and precision, AUC quantifies intrinsic separability across all possible thresholds, and normalized confusion matrices reveal class-specific behavior essential for interpreting diagnostic reliability in breast thermography.

## 3. Experiments

This section presents the experimental evaluation conducted to assess the diagnostic potential of dynamic breast thermography and the performance of machine learning models for classification. The experimentation was structured in two complementary parts.

As a first step, a quantitative analysis of thermal recovery kinetics was performed using dynamic thermographic sequences under cold-stress conditions. This analysis provides a functional characterization of breast tissue by comparing descriptive thermal features and recovery dynamics between cancer and control groups. The results of this stage are presented in Section 3.2.

In the second part, the focus shifts to classification tasks based on multivariate time series representations derived from the thermographic data. Two complementary approaches were implemented: the TSF algorithm and LSTM networks. While TSF offers an interpretable, feature-based view of dynamic thermal behavior, LSTM models capture complex temporal dependencies through sequence learning. Together, these methods provide a comprehensive evaluation of the predictive value of dynamic thermography.

### 3.1. Image Database

Infrared images were obtained from a public database DMR-IR [6]. It has 287 volunteers, of which 244 are reported as healthy (control group), 39 are sick (cancer group), and 4 have an unknown diagnosis. Diagnoses were made in people using mammography and/or biopsy. The database contains infrared images with their associated floating-temperature matrices, digitized mammograms, and clinical data. Frontal images are considered for this analysis.

**Figure 7:**
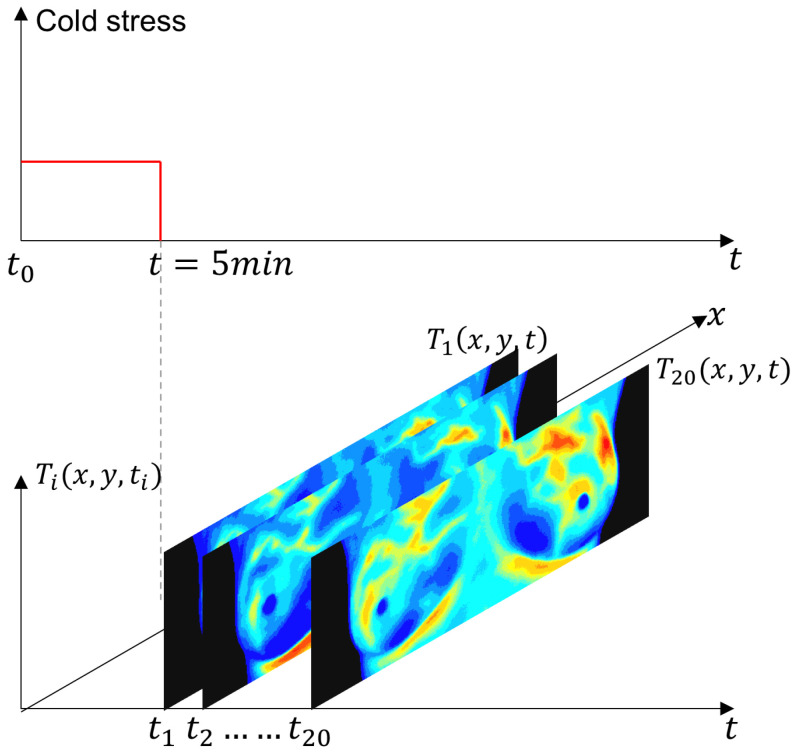
Schematic representation of the experimental protocol for dynamic thermography. The breast region was cooled using an electric fan until the mean temperature reached 30.5 °C or a maximum duration of 5 min was reached. After the thermal stress was removed, a temporal sequence of 20 thermograms was acquired at a uniform sampling interval. The red solid line indicates the applied thermal stress period, while the gray dashed line marks the end of the cooling phase and the start of thermogram acquisition.

The IR images were obtained using the static and dynamic acquisition protocols in an environment between 20 °C and 22 °C, as shown in Figure 7. The steady-state temperature distribution is recorded as a first step. The patient, only in the RoI, is then subjected to a short pulse excitation and cold stress. Thermograms are recorded during the cooling and thermal recovery phases for thermal transient analysis [6].

**Figure 8:**
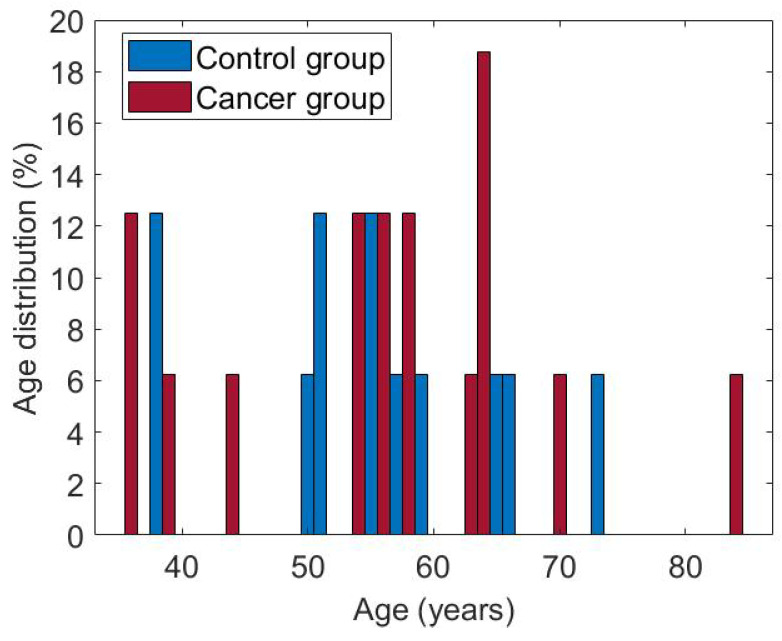
Age of patients in both groups, cancer and control.

The subset of cases used in this research consists of 50 women aged between 36 and 66. Both groups are balanced, with 25 patients each. Figure 8 shows the age distribution for each of them. The cases considered have the complete dynamic sequence of 20 thermograms.

### 3.2. Quantitative Analysis of Dynamic Breast Thermography

**Figure 9:**
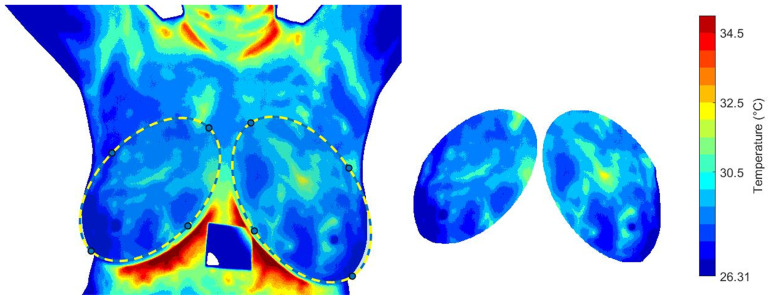
The RoI obtained from the *i*-th thermogram and used for the dynamical analysis of the temperature distribution.

The effect of cooling stress on tissue samples and the subsequent recovery phase is analyzed. Mean, minimum and maximum temperatures, $T_{mean}$, $T_{max}$ and standard deviation, are calculated for the descriptive analysis. The segmentation proposed by [56] is considered. An image mask is used to extract the temperature data from the temperature matrix, see Figure 9. The same RoI selection is applied to each thermogram in the dynamic sequence.

**Figure 10:**
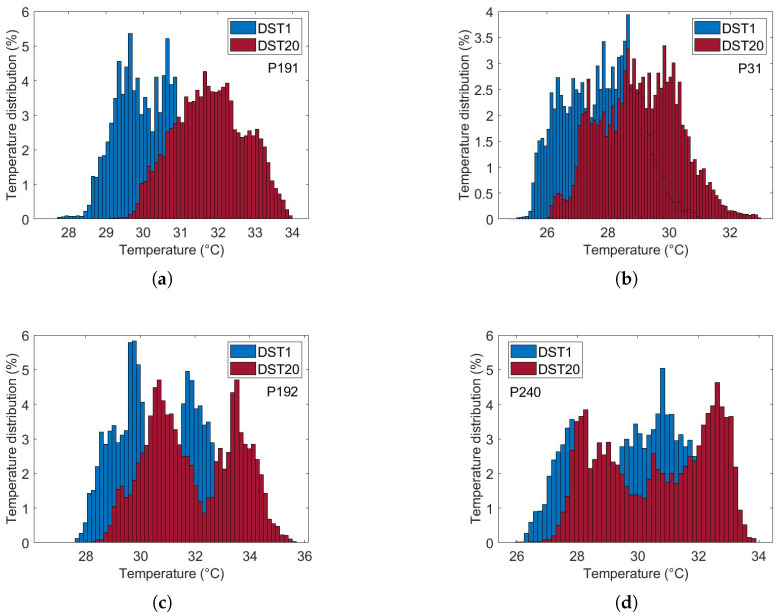
Comparative analysis of dynamic thermogram recovery patterns following cold-stress challenge. (**a**,**b**) Control group showing symmetrical, homogeneous thermal recovery. (**c**,**d**) Cancer group demonstrating asymmetrical rewarming with focal hyper-recovery indicative of tumor angiogenesis and elevated metabolic activity. STD1: immediately post-cooling; STD20: after 5 min (20 sampling intervals).

Figure 10 illustrates the thermal recovery kinetics of breast tissue following cold-stress induction, comparing representative subjects from control and cancer groups. The sequence captures the dynamic evolution from immediate post-stress (STD1) to full recovery after five minutes (SDT20).

The differential recovery patterns are pathognomonic of malignancy-induced physiological alterations. Specifically, the cancer group (c and d) exhibits accelerated rewarming, a direct thermographic correlate of tumor angiogenesis and elevated metabolic activity. In contrast, the control group (a and b) demonstrates slow, homogeneous rewarming, reflecting regulated physiological thermoregulation.

This enhanced thermal contrast under cold-stress conditions improves diagnostic sensitivity by suppressing background thermal noise and accentuating pathological signatures (Table 1). These findings underscore the importance of assessing thermal recovery kinetics rather than relying solely on static thermal patterns.

#### Parameter Estimation

**Figure 11:**
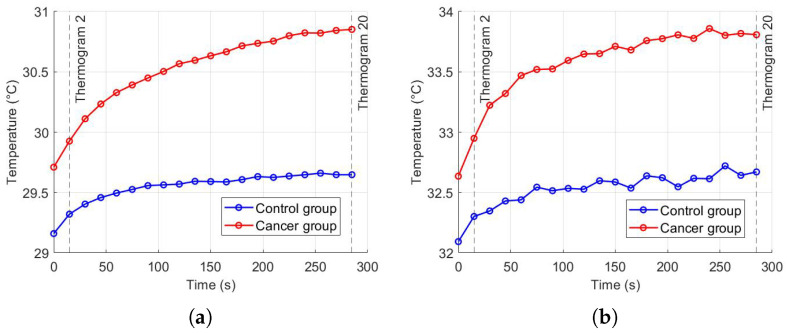
Temperature recovery in the RoI after cold stress: (**a**) $T_{mean}$, (**b**) $T_{max}$.

**Figure 12:**
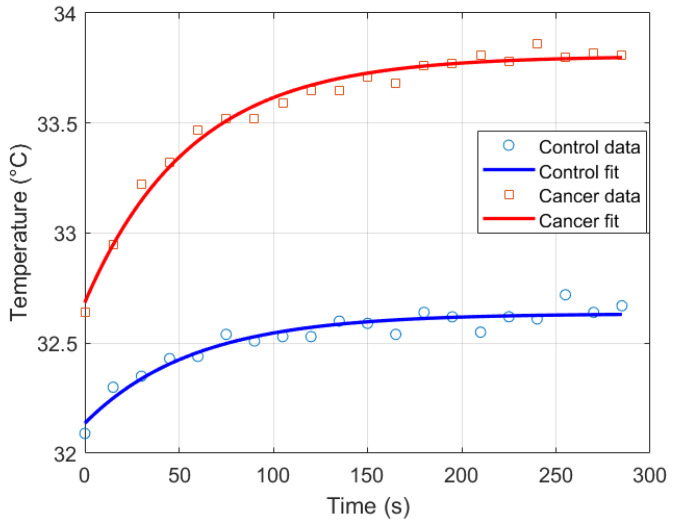
Exponential recovery fit for control and cancer groups.

Thermal recovery kinetics were characterized by fitting the experimental temperature curves to the exponential model described in Section 2.2, allowing extraction of the recovery time constants (τ), initial temperatures ($T_{0}$), and asymptotic temperatures ($T_{\infty }$) for both control and cancer groups. Immediately after cold stress, the cancer group exhibited faster temperature rise in the selected RoI, with the mean temperature ($T_{mean}$) approximately 0.57 °C higher than the control group and the maximum temperature ($T_{max}$) reaching 0.21 °C higher after five minutes (Figure 11). Nonlinear regression was employed to minimize the squared differences between experimental and modeled temperatures, yielding *T*_0_ ≈ 32.14 °C, *T*_∞_ ≈ 32.63 °C, and τ=57.76 s for the control group, and *T*_0_ ≈ 32.69 ° C, *T*_∞_ ≈ 33.80 °C, and τ=56.26 s for the cancer group (Figure 12). The slightly smaller τ observed in cancer tissue indicates faster thermal recovery, consistent with enhanced vascularization and metabolic activity. Descriptive statistics of $T_{mean}$, σ, and $T_{max}$ across 20 thermograms for both groups are summarized in Table 2.

**Figure 13:**
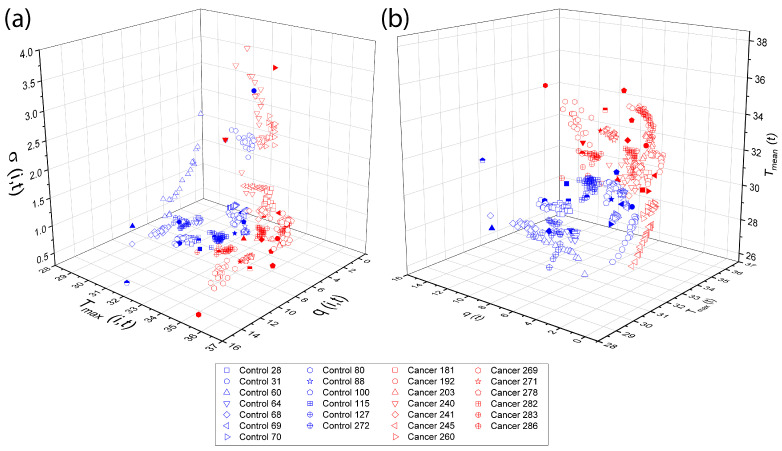
Three-dimensional The overlapping elements do not affect scientific interpretation, as they correspond to different data points within the same feature space. The figure caption was revised to improve clarity and consistency. scattergrams of physiological parameters from the D-I-R model. Scattergrams show clear separation between control and cancer tissue, highlighting the relevance of these features for classification. The classification step make use of the physiological pattern vectors (**a**) $x_{i,t}=${$T_{max}\left(i,t\right),\sigma \left(i,t\right),q\left(i,t\right)$}, (**b**) $x_{i,t}=${$T_{max}\left(i,t\right),T_{mean}\left(i,t\right),q\left(i,t\right)$}.

Physiological parameters used as input features for both TSF and LSTM classifiers were obtained following the procedures described in Section 2.4 and Section 2.5, using the D-I-R fitting methods. These parameters, including q(t), $T_{mean}\left(t\right)$, $T_{max}\left(t\right)$, σ(t), and *d*, quantify the thermal recovery dynamics for each thermogram. Three-dimensional scattergrams (Figure 13) are generated to visualize the correlations and separability between control and cancer tissue, providing a clear rationale for their use as features in the subsequent multivariate classification analysis.

### 3.3. Implementation of the Multivariate TSF Classifier

In this study, we do not classify thermograms directly. Instead, each subject is represented by a multivariate time series that summarizes the temporal evolution of physiologically meaningful descriptors extracted from the RoI of each thermogram. Specifically, every person is described by a 20-point temporal sequence of five features—*q*, $T_{\max}$, $T_{\mathrm{mean}}$, σ, and *d*—computed from the corresponding thermograms in the dynamic recovery protocol. These sequences capture both spatial and temporal aspects of tissue thermal behavior, allowing the TSF classifier to operate on structured physiological time series data rather than raw infrared images.

The TimeSeriesForestClassifier from sktime is used to classify multivariate time series formatted as pandas.Series per variable, without normalization. The dataset included 50 thermograms (25 cancer, 25 control) and was split into training (80%) and test (20%) sets using stratified sampling. Hyperparameters (number of estimators: 100, 200, 300; minimum interval lengths: 3, 5, 7) are optimized via 5-fold stratified cross-validation with parallel computation (n_jobs=−1). The final model is trained on the full training set and evaluated on the test set, reporting Accuracy, F1-score, and AUC.

A 5-fold stratified cross-validation is additionally performed on the entire dataset using the selected hyperparameters. This evaluation provides mean Accuracy, F1-score, and AUC, together with their standard deviations, and enables the computation of an averaged ROC curve with its associated variability. Cross-validation is essential in this context, as the limited dataset size and the nature of time series classification can lead to variability in model performance. By repeatedly training and testing on different data partitions, it yields more reliable estimates of generalization capability and ensures that the reported metrics reflect consistent trends rather than artifacts of a particular train/test split.

#### 3.3.1. Feature Selection Analysis

**Figure 14:**
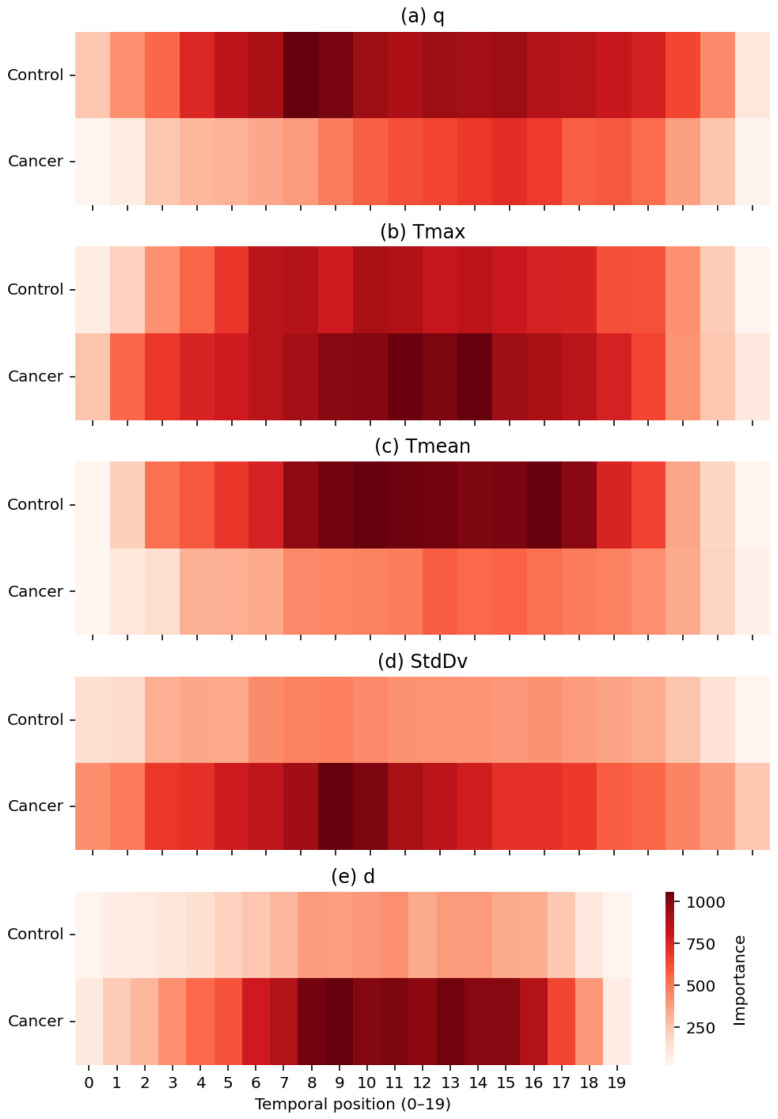
Temporal importance maps for the five thermal descriptors: (**a**) *q*, (**b**) *T_max_*, (**c**) *T_mean_*, (**d**) *StdDv*, and (**e**) *d*. Darker regions indicate higher contribution to the TSF classifier, with cancer cases showing stronger localized patterns in descriptors linked to hyperthermia, heterogeneity, and structural thermal changes.

Figure 14 provides a joint machine-learning and physiological interpretation of how the TSF classifier allocates relevance across temporal positions when using the full set of RoI-derived thermal descriptors. From a modelling perspective, the impurity-based temporal importance profiles reveal the specific time indices within the 20-frame post-stimulus sequence that most strongly influence the ensemble’s decision structure. This offers an intrinsic form of interpretability: TSF relies on localized temporal divergences rather than static summary information, enabling a direct mapping between discriminative features and the temporal dynamics of the underlying thermal response.

Each heatmap quantifies the contribution of temporal positions (1–20) for a single descriptor in the set $\left\{q,T_{\max},T_{\mathrm{mean}},\sigma ,d\right\}$, exposing physiologically meaningful distinctions between healthy and malignant tissue. Heat generation *q* shows higher relevance in control subjects, consistent with more stable metabolic heat transfer in normal breast tissue. In contrast, $T_{\max}$ exhibits pronounced importance peaks in cancer cases, particularly between positions 7–13, reflecting localized hyperthermia driven by tumour-induced angiogenesis. The global descriptor $T_{\mathrm{mean}}$ demonstrates smoother and more uniform importance patterns in controls, whereas the variability descriptor σ highlights increased spatial heterogeneity, an established hallmark of disorganized cancer vasculature. Finally, the depth-related descriptor *d* contributes at specific intervals associated with deeper thermal alterations characteristic of malignant tissue.

Importantly, the temporal structure of these importance profiles is consistent with the thermal recovery model presented in Section Parameter Estimation. The exponential rewarming curves indicate that the greatest physiological divergence between malignant and healthy tissue occurs during the early-to-mid recovery phase, when cancerous regions display a faster warming rate, increased curvature, and a higher steady-state temperature. This physiologically defined critical period of thermal recovery corresponds closely to the interval (approximately positions 8–14) in which the TSF classifier assigns its highest feature importance, most notably for the descriptor *d*, followed by σ and $T_{\max}$. The separation observed in the recovery dynamics therefore provides an independent physiological justification for the temporal locations and descriptors prioritized by the TSF during classification.

Taken together, these results show that the TSF effectively captures discriminative thermal biomarkers that reflect both dynamic and structural physiological processes. The agreement between the temporal importance profiles, the modelled thermal recovery behaviour of breast tissue, and the observed classification performance reinforces the biological and algorithmic plausibility of the TSF decision-making process.

#### 3.3.2. TSF Classifier Results Analysis

Table 3 summarizes the performance of the TSF classifier across the four evaluated feature sets. The AUC values provide a threshold-independent assessment of discriminative ability: all feature sets achieve AUC values above 0.90, indicating excellent separation between healthy and pathological cases. The best-performing configuration, $FS2=\{q,T_{\max},T_{\mathrm{mean}},\sigma \}$, reaches an AUC of 0.976±0.032, approaching near-optimal discrimination. Accuracy and F1-score follow the same trend, reflecting both strong predictive performance and consistent behaviour across folds.

The comparative evaluation of FS1-FS4 highlights the relative contribution of individual descriptors. FS1, corresponding to the full descriptor set, performs strongly, but removing the depth-related feature *d*, FS2, yields a notable improvement in AUC while maintaining stable Accuracy and F1-score. This suggests that *d* introduces additional variance without enhancing discriminative power. Further descriptor reduction in FS3 and FS4 results in progressively lower performance, reflecting the loss of complementary information provided by $T_{\mathrm{mean}}$ and σ, which capture global temperature trends and spatial heterogeneity, respectively.

**Figure 15:**
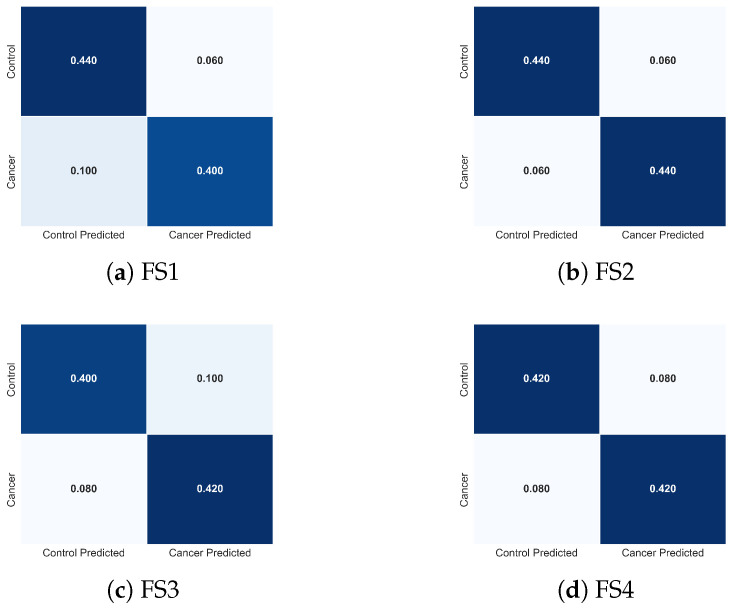
Normalized confusion matrices obtained with the TSF classifier for the four evaluated feature sets: (**a**) FS1, (**b**) FS2, (**c**) FS3, and (**d**) FS4. Each matrix illustrates how the composition of thermal descriptors influences the distribution of classification errors between cancer and control cases, complementing the performance metrics reported in Table 3.

To complement these quantitative results, Figure 15 presents the normalized confusion matrices for FS1-FS4. These visualizations clarify how each descriptor combination modulates class-specific errors. FS2 produces the most balanced confusion structure, with minimal false negatives, the most clinically critical error type in cancer detection. FS1 exhibits slightly increased false negatives, whereas FS3 and FS4 show asymmetric misclassification patterns consistent with the reduced descriptor diversity. These observations provide an intuitive, class-level interpretation of the trends reported in Table 3.

The combined evidence indicates that TSF leverages physiologically meaningful thermal descriptors to differentiate malignant from healthy tissue. Features linked to tumour-induced hyperthermia ($T_{\max}$), metabolic heat evolution (*q*), and spatial heterogeneity (σ) play central roles in classifier performance, while global thermal evolution ($T_{\mathrm{mean}}$) enhances stability and reduces ambiguity in borderline cases. These findings underscore that optimal discrimination arises from integrating temporal, local, and spatial thermal cues, aligning with known biophysical responses of cancerous tissue to cooling and rewarming.

Importantly, the error patterns observed across FS1-FS4 are consistent with the temporal relevance mechanisms described in Section 3.3.1. As shown in the TSF importance maps, the classifier relies on physiologically meaningful temporal divergences, particularly localized hyperthermia, spatiotemporal heterogeneity, and deviations in global thermal recovery, to support its decision-making process. The convergence between numerical performance, confusion-matrix structure, and temporal importance profiles reinforces both the biological plausibility and algorithmic robustness of the TSF classifier.

### 3.4. LSTM-Based Classification

To capture the temporal dependencies in breast thermographic sequences, we implemented a two-layer LSTM network for thermograms classification based on multivariate thermal time series. A multivariate time series represent each person *i*.

$$X_{i}\in R^{M\times V},$$

where M=20 corresponds to the number of thermograms in the dynamic recovery sequence, and V=5 corresponds to the extracted thermal features (as defined in Section 2.4). The original dataset consisted of N=50 patients, evenly split between the cancer (n=25) and control (n=25) groups. Prior to training, all features were globally standardized to ensure numerical stability and uniform scaling across variables.

#### 3.4.1. Data Augmentation

To overcome the limitations of a relatively small dataset, we applied two data augmentation strategies that preserve physiological plausibility:

1. Gaussian noise injection: Random noise with zero mean and standard deviation of 0.05 was added independently to each feature value. This simulates minor sensor variations and slight physiological fluctuations, producing augmented sequences with values realistically deviating by a few hundredths of a degree Celsius.
2. Temporal shifting: Each time series was shifted forward by 1 or 2 frames to account for small variations in the onset of thermal recovery among patients. The missing initial time points were filled by repeating the earliest available measurement, preserving the overall temporal pattern while introducing timing variability.

These augmentations expanded the training dataset by a factor of 3, improving the generalization of the LSTM without compromising physiological interpretability.

**Figure 16:**
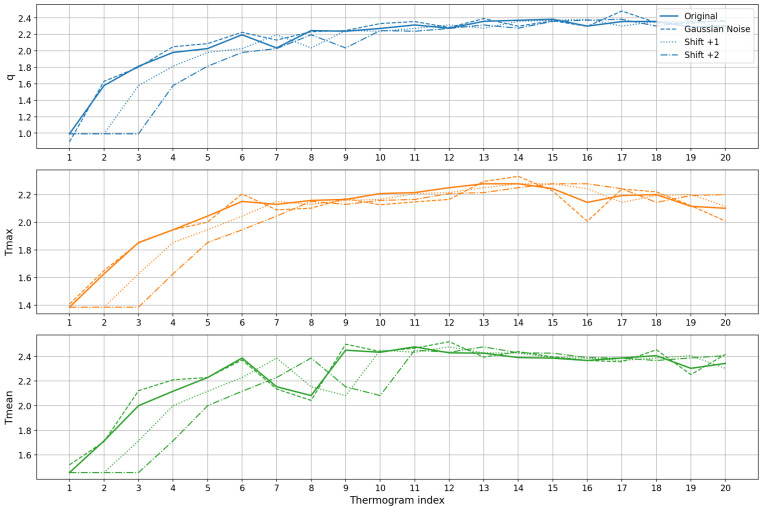
Data augmentation examples for a single patient’s thermal feature time series. Each subplot corresponds to one feature: $q\left(t\right),T_{max}\left(t\right)$, and $T_{mean}\left(t\right)$. The original sequence (solid line) is compared to augmented sequences generated by Gaussian noise (dashed line) and temporal shifts of +1 (dotted line) and +2 (dash-dot line) time steps. The augmentation introduces minor variations in magnitude and timing while preserving the overall thermal recovery pattern, enhancing the model’s ability to learn robust temporal features.

Figure 16 shows representative examples of an original sequence of thermograms and the corresponding augmented sequences generated by Gaussian noise and temporal shifting. The plots show that augmentation introduces subtle variations in magnitude and timing while preserving the overall rewarming pattern, enabling the model to learn more robust temporal features.

#### 3.4.2. *LSTM Architecture and Training*

The LSTM model consisted of two stacked LSTM layers with 64 and 32 memory units, respectively. Batch normalization and dropout layers (rate 0.5) were applied to improve training stability and reduce overfitting. A dense layer with 16 neurons and ReLU activation preceded the final sigmoid output used for binary classification. The model was trained using the Adam optimizer and binary cross-entropy loss, employing early stopping based on validation loss to ensure convergence.

The LSTM model consisted of two stacked LSTM layers with 64 and 32 memory units, respectively. Batch normalization and dropout layers (rate 0.1) were applied to improve training stability and reduce overfitting. A dense layer with 16 neurons and ReLU activation preceded the final sigmoid output used for binary classification. The model was trained using the Adam optimizer and binary cross-entropy loss, employing early stopping based on validation loss to ensure convergence.

Data augmentation was applied as described in Section 3.4.1. This augmentation step was performed only on the training folds and contributed to improving generalization under the limited sample size.

A 5-fold cross-validation protocol was implemented to provide robust and unbiased performance estimates. Normalization was performed independently within each fold to avoid information leakage, after which the augmented sequences were used for training. This procedure ensured a reliable assessment of the model’s generalization across different patient subsets.

In general, the LSTM achieved consistent performance across folds, highlighting its ability to capture relevant temporal dynamics and underscoring the importance of data augmentation and cross-validation when working with small biomedical time series datasets.

#### 3.4.3. *Interpretation of Classification Results*

The results summarized in Table 4 reveal statistically robust behaviour across feature sets and reflect physiologically meaningful patterns associated with thermographic breast analysis. The full descriptor set $\left\{q,T_{\max},T_{\mathrm{mean}},\sigma ,d\right\}$ achieved strong performance, with an AUC of 0.967±0.017, accuracy of 0.940±0.049, and an F1-score of 0.938±0.055. The low variance across folds indicates a stable decision process and suggests that this combination captures the main biophysical characteristics distinguishing cancer from control cases.

From a physiological standpoint, the descriptors encode complementary aspects of tumour-associated thermal behaviour. Metabolic heat generation (*q*) reflects perfusion-driven thermodynamics linked to tumour angiogenesis. Surface temperature descriptors ($T_{\max}$, $T_{\mathrm{mean}}$) capture the magnitude and global distribution of heat transfer from deeper tissue layers. Thermal variance (σ) highlights spatial irregularities typical of disorganized malignant vasculature. Tumour depth (*d*) modulates the amplitude of surface temperature expression through conductive attenuation. Together, these variables form a coherent representation of the spatiotemporal dynamics underlying breast thermogram.

**Figure 17:**
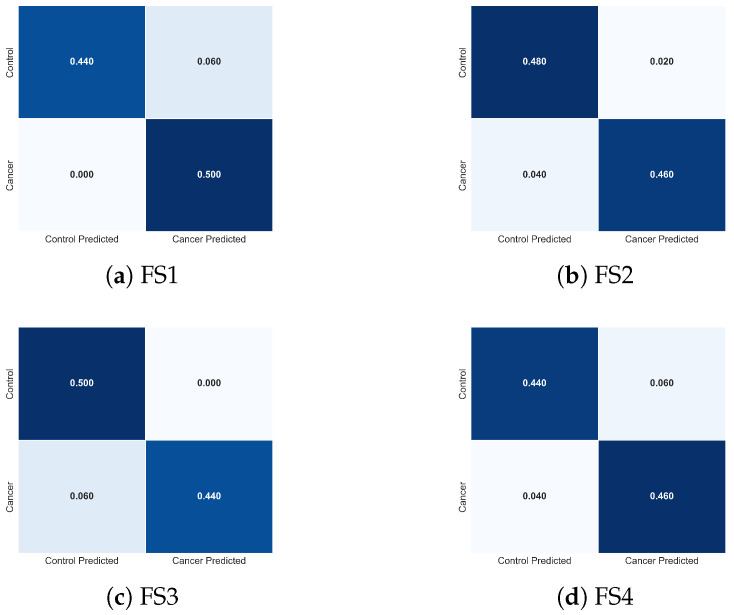
Normalized confusion matrices obtained using the LSTM classifier for the four evaluated feature sets: (**a**) FS1, (**b**) FS2, (**c**) FS3, and (**d**) FS4. Each matrix illustrates how the composition of descriptors influences the distribution of classification errors between cancer and control cases, complementing the performance metrics reported in Table 4.

To further interpret the behaviour of the LSTM classifier, Figure 17 presents the normalized confusion matrices for each feature set. These matrices illustrate how descriptor composition modulates class-specific prediction errors. The full feature set (FS1) yields a symmetric confusion pattern and achieves perfect sensitivity, correctly identifying all cancer cases (0% false negatives), while maintaining a low false-positive rate. This balanced behaviour is desirable in clinical screening, where avoiding missed malignancies is critical.

Removing tumour depth (FS2) produces a confusion matrix nearly identical to that of FS1, confirming that *d* contributes minimal additional discriminative value at the temporal and thermal scales represented in this dataset. Both configurations show equivalent true-positive and true-negative behaviour, consistent with their nearly overlapping performance metrics.

The reduced feature sets (FS3 and FS4) exhibit progressively more asymmetric error patterns. In FS3, the absence of σ results in a modest increase in false negatives (0.06), indicating reduced sensitivity to spatial temperature irregularities. FS4, where $T_{\mathrm{mean}}$ is removed, shows the greatest degradation: despite correctly identifying most cancer cases, this feature set misclassifies a larger proportion of control samples, indicating diminished specificity and reduced model stability. These shifts directly mirror the lower accuracy and higher variance reported for FS4 in Table 4.

Altogether, the confusion matrices demonstrate that the LSTM relies on a physiologically coherent subset of descriptors, particularly *q*, $T_{\max}$, and $T_{\mathrm{mean}}$, to achieve high discriminative performance. The degradation observed when removing $T_{\mathrm{mean}}$ or σ confirms their importance in capturing global thermal elevation and spatial heterogeneity. These visual patterns reinforce the statistical findings and clarify how each feature subset shapes the classifier’s decision boundaries.

In general, the metrics and confusion patterns in Table 4 and Figure 17 demonstrate that integrating dynamic heat information (*q*), maximal and mean temperature descriptors ($T_{\max}$, $T_{\mathrm{mean}}$), and thermal variability (σ) provides a physiologically grounded and diagnostically meaningful representation of tumour-associated thermal behaviour. These descriptors enable the LSTM to capture the underlying spatiotemporal structure of the thermal response, resulting in high discriminative performance and low fold-to-fold variability for the more complete feature sets.

#### 3.4.4. Comparison Between TSF and LSTM Feature Behaviours

A direct comparison of the TSF and LSTM classifiers reveals a consistent yet mechanistically distinct pattern in how each model exploits the physiological descriptors. TSF operates on interval-based summary statistics, mean, standard deviation, and slope, which are computed over randomly sampled temporal segments. Because TSF never processes the raw temporal trajectory, it depends on σ as an explicit descriptor to quantify spatial–thermal heterogeneity. This reliance is evident in the performance drop observed when σ is removed (FS3) and in the more asymmetric confusion profile associated with this feature set, indicating diminished sensitivity to short-range fluctuations and local irregularities. In contrast, the LSTM processes the full sequence of $T_{\max}\left(t\right)$ and $T_{\mathrm{mean}}\left(t\right)$ at every time step, allowing its recurrent gating mechanisms to implicitly learn heterogeneity patterns, such as abrupt thermal surges, nonlinear recovery rates, and deviations from smooth rewarming trajectories, without requiring σ as an explicit input. Consequently, the LSTM preserves high AUC, accuracy, and F1-score even when σ is excluded, and its confusion matrices remain nearly unchanged across feature sets. In summary, these results highlight the complementary behavior of both models—TSF providing transparent, statistic-driven detection of localized heterogeneity, and LSTM capturing more complex nonlinear dependencies embedded in the dynamic thermogram sequence, offering a consistent physiological interpretation across modeling paradigms.

### 3.5. Statistical Analysis Results

A non-parametric comparison of classifier performance across feature sets was conducted to determine whether the choice of thermal descriptors extracted from the RoI influenced the discriminative capability of the models. The Friedman test results, summarized in Table 5, show that neither the LSTM nor the TSF classifier exhibited statistically significant differences across the evaluated feature sets for any performance metric (AUC, accuracy, or F1-score). For all cases, the corresponding *p*-values were greater than the 0.05 significance threshold, indicating that the null hypothesis of equal median ranks could not be rejected. Although numerical differences were observed among feature sets, these variations were not consistent across validation folds and did not reach statistical relevance. This outcome suggests that the temporal thermal descriptors derived from the RoI, including *q*, $T_{\max}$, $T_{\mathrm{mean}}$, σ, and *d*, provide comparable discriminatory information when used individually or in combination within both classifier architectures.

**Figure 18:**
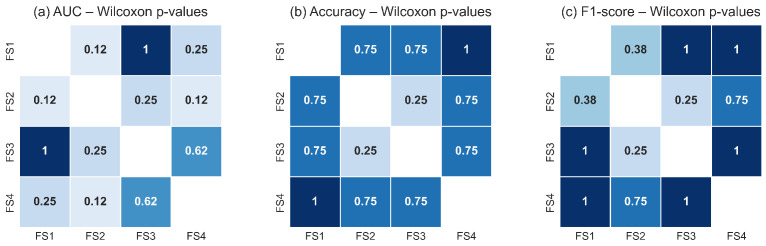
Pairwise Wilcoxon signed-rank *p*-values for the TSF classifier across the four evaluated feature sets for: (**a**) AUC, (**b**) accuracy, and (**c**) F1-score. The uniformly high *p*-values indicate that none of the feature-set pairs exhibits statistically significant differences (*p* ≥ 0.05). Darker tones represent higher *p*-values, reinforcing the conclusion that TSF performance remains statistically equivalent across all evaluated feature configurations.

**Figure 19:**
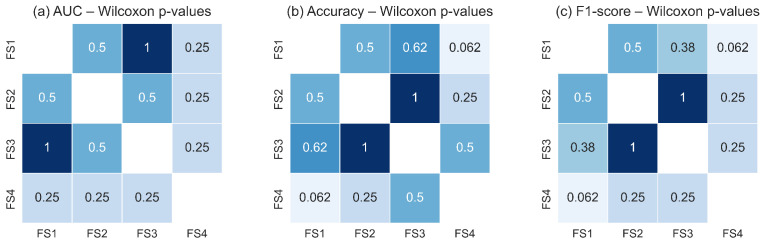
Pairwise Wilcoxon signed-rank *p*-values for the LSTM classifier across the four evaluated feature sets for: (**a**) AUC, (**b**) accuracy, and (**c**) F1-score. As with the TSF classifier, the consistently high *p*-values indicate no statistically significant pairwise differences (*p* ≥ 0.05). Darker shading corresponds to higher *p*-values, confirming the statistical equivalence of feature sets in terms of LSTM performance.

Figure 18 and Figure 19 illustrate the pairwise Wilcoxon signed-rank *p*-values for the TSF and LSTM classifiers, respectively. The heatmaps show uniformly high *p*-values across all pairwise comparisons of feature sets for each metric, with no comparison falling below the significance threshold. The darker shading observed throughout the heatmaps reflects the predominance of large *p*-values, confirming the absence of measurable statistical differences in classification performance between feature-set pairs. These results corroborate the findings of the Friedman test and reinforce the conclusion that all evaluated feature sets capture essential RoI level thermal dynamics with similar discriminative utility.

Taken together, the statistical analyses demonstrate that the classifiers are robust to variations in the selected feature subsets. Whether they include only global RoI descriptors ($q,T_{\max},T_{\mathrm{mean}}$) or also incorporate variability (σ) and structural depth information (*d*), the resulting performance remains statistically equivalent. This suggests that the malignant and non-malignant thermal patterns within the RoI are sufficiently captured by the core thermal descriptors, and that adding additional features does not significantly alter the decision boundaries learned by either model. This stability across classifiers and feature sets underscores the reliability of the RoI-based thermal signal as a basis for discrimination in breast thermography.

## 4. Conclusions

Dynamic infrared thermography, combined with computational modeling and machine learning, provides a robust and physiologically meaningful framework for early breast cancer detection. The key findings of this study can be summarized as follows:

- Quantitative analysis under cold-stress conditions revealed distinct recovery patterns between groups: malignant tissue exhibited accelerated, focal rewarming and increased asymmetry, whereas healthy tissue showed slow and homogeneous recovery.
- The reduction in the thermal recovery constant τ observed in cancer cases confirms faster heat restoration, reflecting enhanced vascularization and elevated metabolic activity.
- The TSF classifier provided interpretable interval-based insights, identifying temporal regions where localized heterogeneity and tumour-associated thermal fluctuations were most discriminative.
- LSTM networks effectively captured sequential dependencies across the 20 thermograms, modeling the full thermal recovery trajectory and achieving the highest overall classification performance.
- Across both classifiers, physiologically informed descriptors, particularly *q*, $T_{\max}$, and $T_{\mathrm{mean}}$, combined with spatial heterogeneity (σ), consistently emerged as the most relevant predictors of malignancy.

Overall, this work demonstrates that integrating dynamic thermography with multivariate time series modeling significantly enhances diagnostic sensitivity and specificity. The proposed approach supports non-invasive, patient-specific profiling of breast tissue, offering a clinically meaningful complement to conventional imaging techniques and enabling earlier, more reliable identification of tumour-associated thermal signatures.

To contextualize the relevance of this methodology within current thermography-based classification strategies, Table 6 summarizes the main methodological differences between conventional image-based deep learning pipelines and the physiological time series framework proposed here. The comparison highlights that the combined LSTM–TSF architecture achieves competitive performance while maintaining interpretability and requiring substantially fewer annotated samples—an important advantage in biomedical applications where large datasets are often unavailable.

## Figures and Tables

**Table 1:** Comparative Thermogram Features Following Cold-Stress Induction.

Feature	Control Group	Cancer Group
Recovery Dynamics	Slow, homogeneous rewarming	Rapid, focal hyper-recovery
Thermal Symmetry	High bilateral symmetry	Significant asymmetry
ΔT	<1.0 °C between breasts	>2.0 °C in lesion area
Hot Spots	Absent	Present

**Table 2:** Descriptive statistics of $T_{mean}$, σ, and $T_{max}$ for control and cancer groups across 20 thermograms.

	Control Group			Cancer Group		
	$T_{\mathrm{mean}}$	σ	$T_{\max}$	$T_{\mathrm{mean}}$	σ	$T_{\max}$
T1	29.16	0.9024	32.09	29.71	0.9252	32.64
T2	29.32	0.9119	32.30	29.93	0.9524	32.95
T20	29.65	0.9789	32.67	30.85	1.0264	33.81

**Table 3:** Performance of the multivariate TSF classifier for different descriptor combinations. AUC, Accuracy, andbF1-score are reported with their standard deviations and reflect the ability of each feature set to discriminate cancer from control cases.

Feature Set	AUC	Accuracy	F1-Score
$FS1=\{q,T_{\max},T_{\mathrm{mean}},\sigma ,d\}$	0.936±0.048	0.86±0.080	0.858±0.079
$FS2=\{q,T_{\max},T_{\mathrm{mean}},\sigma \}$	0.976±0.032	0.86±0.080	0.861±0.067
$FS3=\{q,T_{\max},T_{\mathrm{mean}}\}$	0.944±0.032	0.820±0.075	0.828±0.061
$FS4=\{q,T_{\max},\sigma \}$	0.916±0.065	0.840±0.049	0.836±0.058

**Table 4:** Performance metrics (AUC, accuracy, and F1-score) obtained for the different feature sets evaluated in the LSTM classifier. The features include: *q*, $T_{\max}$ and $T_{\mathrm{mean}}$, σ and *d*. Results are expressed as mean ± standard deviation across the five cross-validation folds.

Feature Set	AUC	Accuracy	F1-Score
$\left\{q,T_{\max},T_{\mathrm{mean}},\sigma ,d\right\}$	0.967 ± 0.017	0.940±0.049	0.938±0.055
$\left\{q,T_{\max},T_{\mathrm{mean}},\sigma \right\}$	0.992±0.017	0.940±0.049	0.945±0.045
$\left\{q,T_{\max},T_{\mathrm{mean}}\right\}$	0.992 ± 0.017	0.940±0.049	0.937±0.052
$\left\{q,T_{\max},\sigma \right\}$	0.909 ± 0.130	0.900±0.089	0.911±0.075

**Table 5:** Friedman test results comparing the performance of the LSTM and TSF classifiers across the four evaluated feature sets for AUC, accuracy, and F1-score. In all cases, the *p*-values exceeded the 0.05 threshold, indicating no statistically significant differences among feature sets for either classifier.

Metric	LSTM			TSF		
	$\chi^{2}$	* p * -Value	Significance	$\chi^{2}$	* p * -Value	Significance
AUC	5.4000	0.1447	Not significant	7.1053	0.0686	Not significant
Accuracy	2.1316	0.5456	Not significant	2.5862	0.4599	Not significant
F1-score	2.3846	0.4965	Not significant	2.9118	0.4054	Not significant

**Table 6:** Methodological comparison between image-based deep learning approaches and the proposed physiological time series framework.

Aspect	CNN/Transformer Thermography	Proposed LSTM–TSF Framework
Input type	Raw thermographic images (static or multi-view)	Physiological time series extracted from a RoI (20 frames)
Feature representation	Pixel-level texture, contrast, deep spatial features	q(t), $T_{\max}\left(t\right)$, $T_{\mathrm{mean}}\left(t\right)$, σ(t), *d*
Temporal modeling	Limited unless using video transformers or 3D CNNs	Direct temporal modeling via LSTM/TSF
Interpretability	Low; relies on saliency/Grad-CAM techniques	High; descriptors have explicit physiological meaning
Data requirements	Large annotated datasets (hundreds–thousands)	Effective even with small-sample datasets (50 subjects)
Performance range	AUC/ACC: 0.90–0.98	AUC up to 0.98 (best configurations)

## Data Availability

The thermograms used in this study were obtained from the publicly available DMR-IR dataset. The multivariate time series derived from these thermograms were generated by the authors and are available from the corresponding author upon reasonable request.
